# Crop production and nitrogen use in European cropland and grassland 1961–2019

**DOI:** 10.1038/s41597-021-01061-z

**Published:** 2021-10-29

**Authors:** Rasmus Einarsson, Alberto Sanz-Cobena, Eduardo Aguilera, Gilles Billen, Josette Garnier, Hans J. M. van Grinsven, Luis Lassaletta

**Affiliations:** 1 Department of Space, Earth and Environment, Chalmers University of Technology, Gothenburg, Sweden; 2 ETSI Agronómica, Alimentaria y de Biosistemas, CEIGRAM-Dpto. Producción Agraria Universidad Politécnica de Madrid, Madrid, Spain; 3 ETSI Agronómica, Alimentaria y de Biosistemas, CEIGRAM-Dpto. de Quimica y Tecnología de los Alimentos Universidad Politécnica de Madrid, Madrid, Spain; 4 Sorbonne Université CNRS EPHE UMR Metis, 7619 Paris, France; 5 PBL Netherlands Environmental Assessment Agency, The Hague, the Netherlands

**Keywords:** Element cycles, Agroecology

## Abstract

This paper presents EuropeAgriDB v1.0, a dataset of crop production and nitrogen (N) flows in European cropland 1961–2019. The dataset covers 26 present-day countries, detailing the cropland N harvests in 17 crop categories as well as cropland N inputs in synthetic fertilizers, manure, symbiotic fixation, and atmospheric deposition. The study builds on established methods but goes beyond previous research by combining data from FAOSTAT, Eurostat, and a range of national data sources. The result is a detailed, complete, and consistent dataset, intended as a basis for further analyses of past and present agricultural production patterns, as well as construction of scenarios for the future.

## Abstract (summary)

**Table:** 

Measurement(s)	agricultural process
Technology Type(s)	Survey
Factor Type(s)	year • geographic location
Sample Characteristic - Environment	agriculture
Sample Characteristic - Location	Europe

Machine-accessible metadata file describing the reported data: 10.6084/m9.figshare.14893077

## Background & Summary

European agriculture has changed dramatically in the last century. Underlying the overall postwar trend of increasing productivity is a complex mix of structural changes. Some regions have continously intensified while others have extensified or abandoned agricultural land^1,2^. Specialization has been a strong trend, visible on subnational as well as national and continental scale^3–6^. The environmental impacts of agriculture, including climate change, air and water pollution, and biodiversity loss, have intensified too—in many areas at an alarming pace—but also in this aspect the development has been heterogeneous^7–9^.

In order to fully understand Europe’s recent agricultural history, and to make informed decisions about its agricultural future, there is a need for an accurate and detailed quantitative picture of these developments. This study contributes to that picture specifically by examining European agricultural flows of nitrogen (N), a key driver of both agricultural productivity and a range of adverse environmental effects^7,10,11^.

This paper presents EuropeAgriDB v1.0^12^, a dataset describing crop production and N flows in European cropland 1961–2019. The dataset is intended as a basis for further analyses of past and present agricultural production patterns, as well as construction of scenarios for the future. Such analyses are particularly relevant in Europe, where agricultural N losses have caused substantial economic and environmental damages and, in response, a number of policies have been launched in the last decades to monitor and control N pollution^13–15^. Among these policies is the recent EU Farm to Fork Strategy^16^, which aims to reduce nutrient losses by at least 50% and fertilizer use by at least 20% by 2030. A first step towards this challenging target is to know the departure point as accurately as possible.

**Fig. 1:**
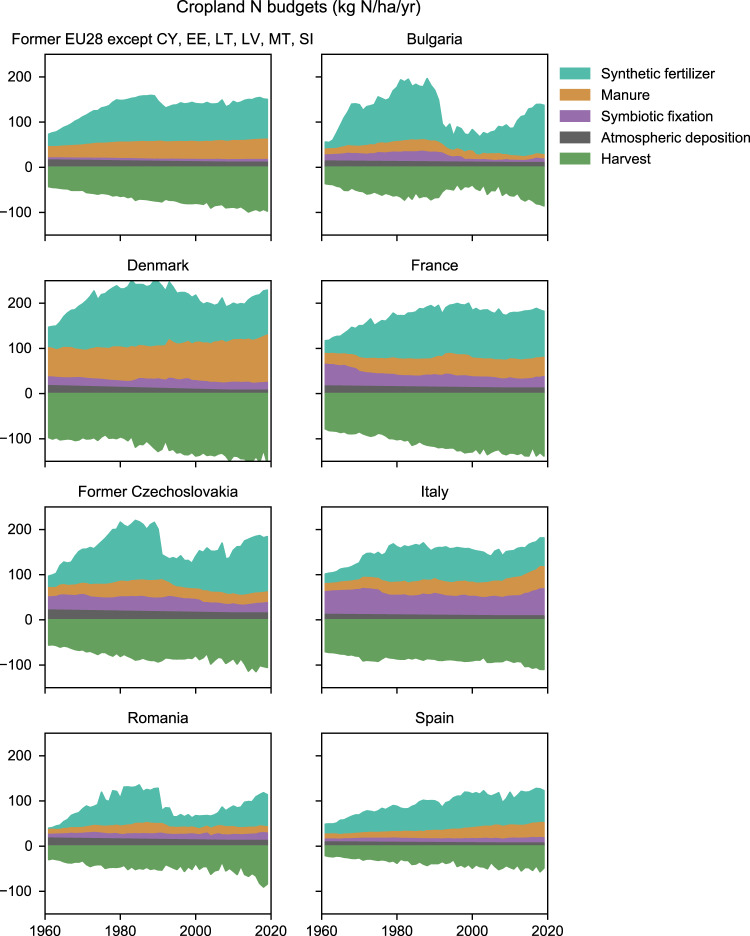
Cropland N budgets for a selection of countries in this study. The top left panel shows results for the 22 present-day countries which this study covers 1961–2019.

This study is based on established methods but goes beyond previous research by combining a range of data sources into a more comprehensive, consistent, and detailed picture of agricultural N flows in Europe than previously available. In particular, we build here on methods developed by Lassaletta *et al*.^17,18^ to estimate global cropland N budgets, accounting for harvested N as well as N inputs in the form of manure, synthetic fertilizers, symbiotic fixation, and atmospheric deposition, based primarily on FAOSTAT data. Here, by narrowing the geographical scope to Europe, we are able to increase the level of detail considerably. Examples of cropland N budgets are shown in Fig. 1. Another relevant dataset to mention here is the agricultural nutrient budgets maintained by Eurostat and the OECD. The Eurostat/OECD nutrient budgets differ from this study in two important aspects: (1) this study starts in 1961, whereas the Eurostat/OECD budgets start around 1985–1990; and (2) this study establishes soil surface N budgets for cropland (excluding permanent grassland), whereas Eurostat/OECD report land N budgets for the entire agricultural area^19,20^. Our aim is not to supplant either of the above-mentioned datasets, but to complement them.

**Fig. 2:**
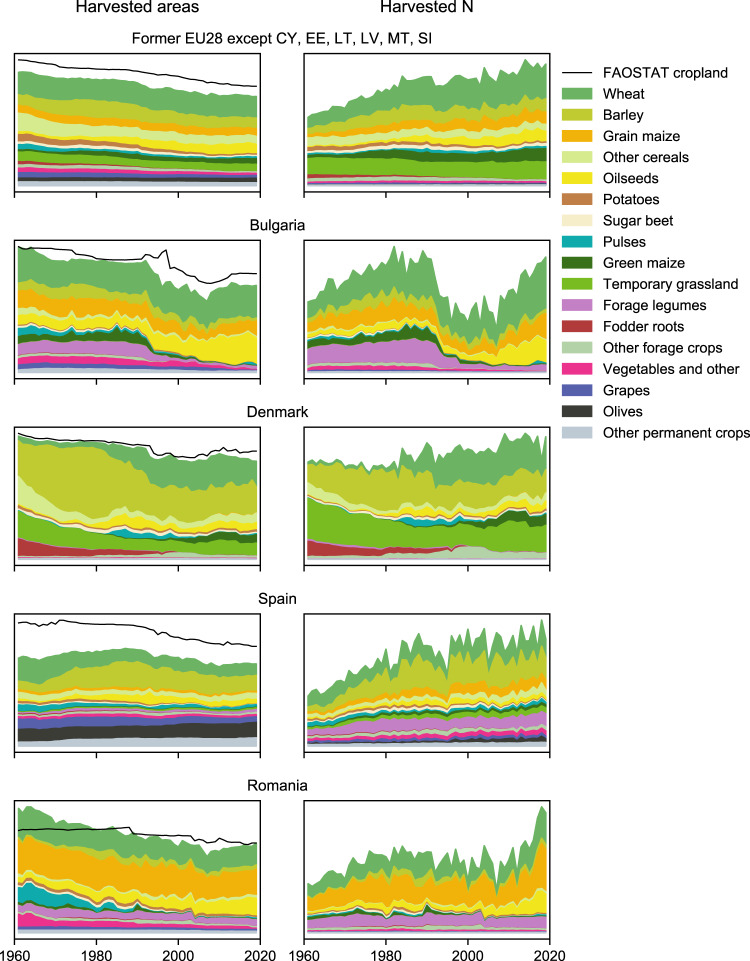
Crop areas and crop N harvests in a selection of countries in this study. The top left panel shows results for the 22 present-day countries which this study covers 1961–2019.

A key contribution of this study is the scope and detail of crop production, in particular fodder crops such as temporary grassland, green maize, and forage legumes. Fodder crops can play an important role in N budgets, as stressed by Zhang *et al*.^21^ in an intercomparison of 13 global datasets, both because fodder crops sometimes contribute substantially to total crop N output and because forage legumes introduce non-negligible amounts of N through symbiotic fixation. For these crops, we have combined the Eurostat crop production statistics database with a range of national databases and other sources. For other arable and permanent crops, we follow Lassaletta *et al*.^17^, using the FAOSTAT database which apart from fodder crops offers the longest and most complete time series of crop production. We report crop production data for 17 crop categories (see Fig. 2), providing a solid basis for understanding how crop mix and productivity have varied over time.

**Fig. 3:**
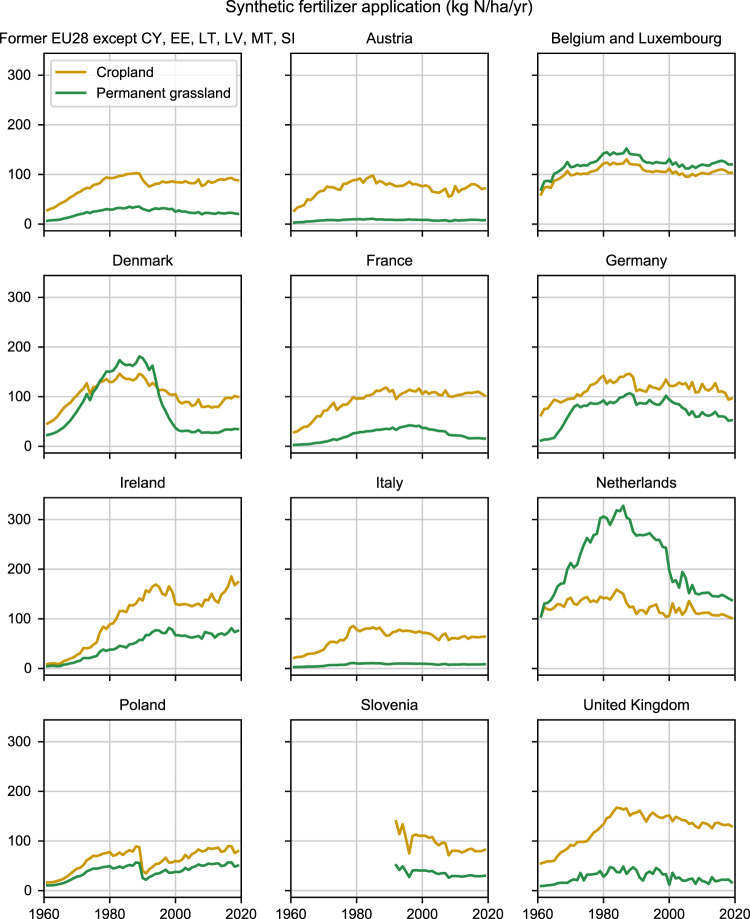
Average rates of synthetic N fertilizer applied to cropland and permanent grassland. The figure shows all countries where more than 3% of the cumulative N fertilizer use has been applied to permanent grassland. The top left panel shows results for the 22 present-day countries which this study covers 1961–2019.

A second key contribution of this study is the increased detail in the allocation of synthetic N fertilizer application between cropland and permanent grassland (see Fig. 3). We have assembled a comprehensive dataset and devised a rigorous method to process it. The result is the most comprehensive and consistent estimate of the allocation of synthetic N fertilizer between cropland and permanent grassland to date.

Geographically, the dataset presented here covers the 26 present-day countries Austria, Belgium, Bulgaria, Croatia, Czechia, Denmark, Estonia, Finland, France, Germany, Greece, Hungary, Ireland, Italy, Latvia, Lithuania, Luxembourg, the Netherlands, Poland, Portugal, Romania, Slovakia, Slovenia, Spain, Sweden, and the United Kingdom, i.e., the countries in the former EU28 except Cyprus and Malta. Most of these countries are covered for the period 1961–2019 (see Method section for details).

**Fig. 4:**
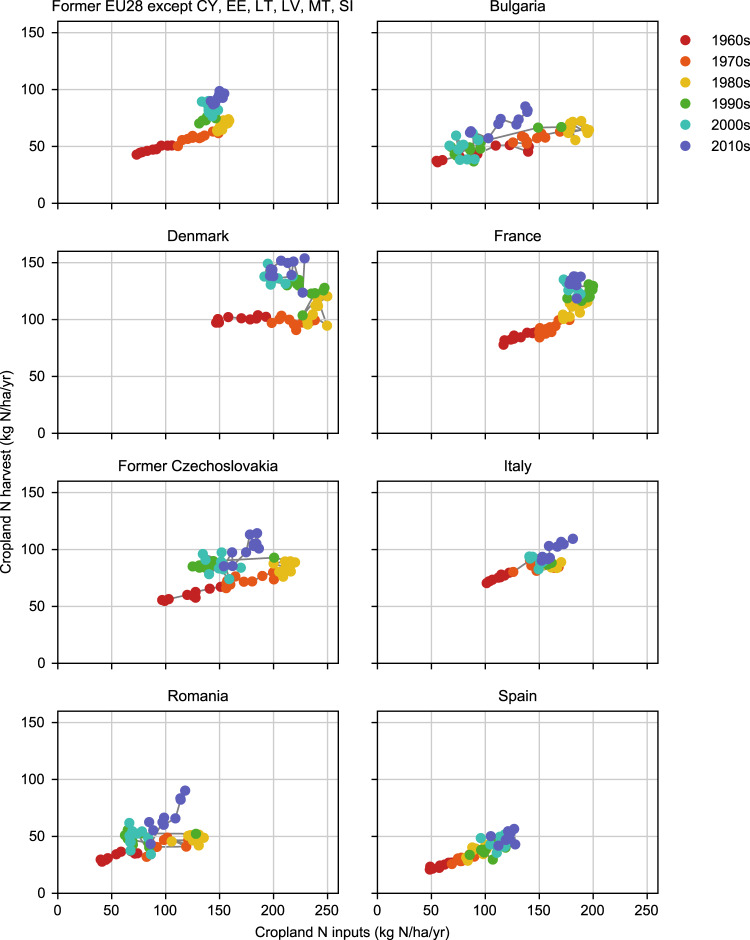
Trajectories in terms of total cropland N harvest and total N input rate to cropland (synthetic fertilizer, manure, symbiotic fixation, and atmospheric deposition). The top left panel shows results for the 22 present-day countries which this study covers 1961–2019.

This paper will hopefully find reuse value in two ways. First, the results provide a consistent, complete, and detailed picture of N use in cropland, which can serve as a basis for future assessments of agricultural productivity, efficiency, and N losses. See Fig. 4 for an example of different trajectories that can be quantified using the results of this paper. Second, the input data collated from various literature sources, and the detailed descriptions in this paper of how we used them, are useful for researchers who wish to further improve on similar descriptions of past and present agricultural production in Europe, for example in the current context of the implementation of the Farm to Fork strategy. In summary, the dataset presented here will be useful both to understand Europe’s recent agricultural history and to make informed decisions about its future.

## Methods

### Method and data overview

The main objective of this study was to assemble a consistent and detailed dataset of annual cropland N budgets in Europe 1961–2019. To this end, we collated a range of different datasets. The FAOSTAT database is the primary data source, providing the longest and most complete data on crop and livestock production with global coverage. However, limiting our scope to the former EU28 allows us to leverage the Eurostat database which in certain respects has more comprehensive coverage. The FAOSTAT and Eurostat databases are both based on national statistics, but have several differences in scope and statistical nomenclature. Therefore, as this paper will demonstrate, the two databases in combination provide a richer picture than either one separately. In addition to these international datasets, we have compiled data from several national statistical databases and yearbooks as well as other literature sources. The main international databases used in this study are listed in Table 1. Remaining data sources are referred to in the text.

**Table 1:** Main international databases used.

Dataset	Version date	Refs.
FAOSTAT crop production, normalized	2021-01-13	^24,181^
FAOSTAT land use, normalized	2021-01-13	^22^
FAOSTAT fertilizer nutrients, 1961-present, normalized	2021-01-13	^83^
Eurostat Annual Crop Statistics (ACS) (apro_cpnh1, apro_cpsh1)	2020-12-14 23:00	^23,28^
Eurostat Annual Crop Statistics (ACS), historical (apro_cpnh1_h)	2020-02-27 11:00	^23,28^
Eurostat fertilizer consumption (aei_fm_usefert)	2021-01-04 11:00	^84^
Eurostat sales of manufactured fertilizers (aei_fm_manfert)	2020-09-08 23:00	^85^
Eurostat Farm Structure Survey (FSS) 2005–2016 (ef_lus_allcrops)	2019-09-13 23:00	^29^
Eurostat Farm Structure Survey (FSS): farmland 1990–2007 (ef_lu_ovcropaa)	2019-05-06 23:00	^29^
IFA fertilizer consumption	2021-01-28	^86^

For the FAOSTAT and IFA sources the version date refers to the download date, and for the Eurostat sources it refers to the publication date in Eurostat’s bulk download facility.

The focus of this study is cropland, defined as land under arable crops (including temporary grassland) and permanent crops (e.g., fruit orchards). Cropland does not include permanent grasslands, defined by both FAOSTAT and Eurostat as grassland lasting for more than five years^22,23^. In some cases, however, the results obtained in this work have obvious relevance for permanent grassland as well. For example, our method to estimate synthetic N fertilizer input to cropland also produces an estimate of input to permanent grassland.

As explained above, this study covers a territory which today comprises 26 countries. The territory is covered in the period 1961–2019 with the exceptions of Croatia, Estonia, Latvia, Lithuania, and Slovenia, which all gained independence in 1991/1992 and due to data limitations are covered in the period 1992–2019. We follow the nomenclature and regional divisions of the FAOSTAT database. For the period 1961–1999, the FAOSTAT database reports Belgium-Luxembourg as one unit, but since 2000 it reports Belgium and Luxembourg separately. Data are reported for Czechoslovakia 1961–1992, and separately for Czechia and Slovakia since 1993. For the whole period 1961–2019, data are reported for Germany as one unit, the territory known as Germany since the 1990 reunification. In some cases, the FAOSTAT regional nomenclature differs from other data sources—notably, the Eurostat database covers Belgium and Luxembourg separately since the start in 1955, and only West Germany until 1989—and to reconcile such differences we have collected additional data and calculated area-weighted or quantity-weighted averages as appropriate to the extent possible. To facilitate further analyses, we also provide merged 1961–2019 results for Belgium and Luxembourg and former Czechoslovakia. Throughout the paper, we loosely refer to these geographical areas as “countries”.

The remainder of this Method section describes and motivates the data and methods used to estimate the following quantities:

- Harvested areas and harvested crop N in 17 categories of arable and permanent crops.
- Symbiotic N fixation in pulses and forage legumes on cropland.
- Areas of cropland, permanent grassland, and cropland in use.
- Synthetic N fertilizer use, partitioned between cropland and permanent grassland.
- Manure N flows: excreted N, partitioned into grazing and in-house excretion; losses of N (mainly ammonia) from manure management in animal houses and manure storage facilities; quantities of manure N that finally reach cropland and permanent grassland from grazing animals or through field application.
- Atmospheric N deposition to cropland and permanent grassland.

**Fig. 5:**
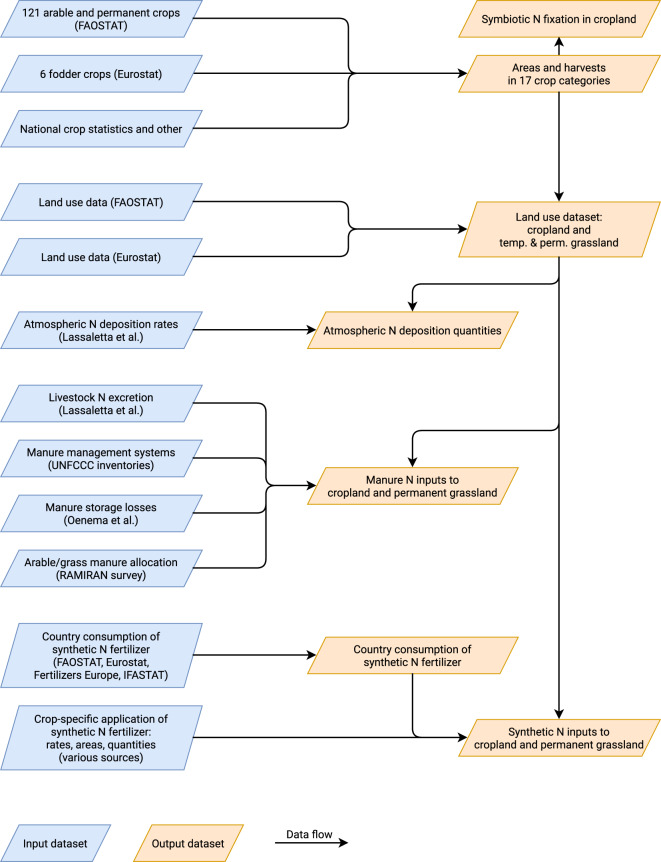
Overview of the main data sources and results of this study. Major input datasets colored blue. Major derived results colored orange.

Figure 5 gives a high-level overview of the main data sources, transformation steps, and results.

Table 2 gives a list of symbols and abbreviations.

**Table 2:** This list covers all non-standard abbreviations and symbols used in more than one subsection of the paper or in the data record.

*A*	Variable name referring to crop or land areas, e.g., *A*_C_ for cropland area.
ACS	Annual Crop Statistics (Eurostat dataset).
C	Used as a subscript to denote cropland, e.g., *A*_C_.
C − TG	Used as a subscript to denote cropland excluding temporary grassland, e.g., *A*_C−TG_.
CLRTAP	Convention on Long-Range Transboundary Air Pollution
EFMA	The European Fertilizer Manufacturers Association. (Today known as Fertilizers Europe.)
EU27	Used here to refer to the former European Union 2007–2013, having 27 member states (before Croatia joined to form EU28).
EU28	The European Union 2013–2020, with 28 member states.
EuropeAgriDB	The name of the dataset described in this study.
Eurostat	The statistics office of the EU.
FAO	Food and Agriculture Organization of the United Nations.
FAOSTAT	FAO’s statistics organization.
FSS	Farm Structure Survey, Eurostat’s triennial survey of agriculture in the EU.
G0000	Eurostat crop code “Plants harvested green from arable land”
G1000	Eurostat crop code “Temporary grasses and grazings”
G2000	Eurostat crop code “Leguminous plants harvested green”
G2100	Eurostat crop code “Lucerne” (alfalfa)
G2900	Eurostat crop code “Other leguminous plants harvested green n.e.c.”
G3000	Eurostat crop code “Green maize”
G9000	Eurostat crop code “Other plants harvested green from arable land”
G9100	Eurostat crop code “Other cereals harvested green (excluding green maize)”
G9900	Eurostat crop code “Other plants harvested green from arable land n.e.c.”
Gg	Gigagram = 10^9^ g = 10^6^ kg = thousand metric tonnes.
IFA	The International Fertilizer Association.
kha	Thousand (10^3^) hectares.
Mha	Million (10^6^) hectares.
PG	Used as a subscript to denote permanent grassland, e.g., *A*_PG_.
PGf	Used as a subscript to denote fertilized permanent grassland, e.g., *A*_PGf_.
*Q*	Variable name referring to (annual) fertilizer quantities expressed, e.g., in Gg N/yr.
*R*	Variable name referring to fertilizer rates expressed, e.g., in kg N/ha/yr.
R0000	Eurostat crop code “Root crops”
R1000	Eurostat crop code “Potatoes (including seed potatoes)”
R2000	Eurostat crop code “Sugar beet (excluding seed)”
R9000	Eurostat crop code “Other root crops n.e.c.”
TG	Used as a subscript to denote temporary grassland, e.g., *A*_TG_.
TGf	Used as a subscript to denote fertilized temporary grassland, e.g., *A*_TGf_.
UNFCCC	United Nations Framework Convention on Climate Change
*Y*	Variable name referring to crop N yields expressed, e.g., in kg N/ha/yr.

In addition, all abbreviations and symbols are defined on their first mention throughout the text.

The input data and results described in this paper as well as source code for all the calculations have been archived as a public data record^12^.

### Crop areas and harvests: overview

We combined a range of data sources to estimate crop areas and N harvests in 17 crop categories.

For most arable and permanent crops, we used crop harvests and areas from the FAOSTAT database (see Table 1).

The only major crops missing from the FAOSTAT database are fodder crops such as temporary grassland, forage legumes, cereal crops harvested green, and fodder roots and cabbages^24^. For these fodder crops, we instead used data from Eurostat’s Annual Crop Statistics (ACS) (see Table 1) and a range of other sources discussed in detail below. The few data on green fodder crops reported in FAOSTAT’s database (only green maize) were excluded to avoid any double-counting.

**Fig. 6:**
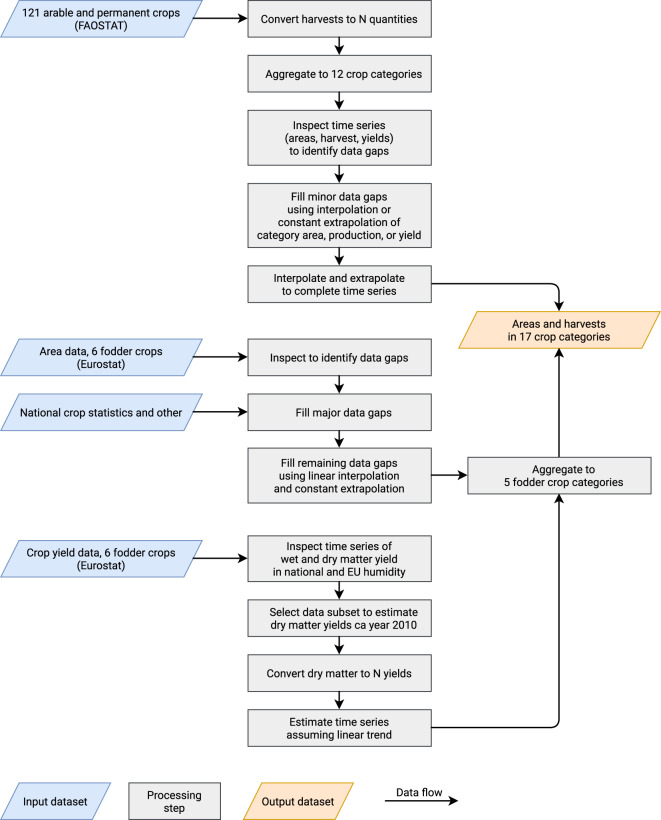
Illustration of main data sources and transformation steps used to estimate areas and N harvests in 17 crop categories. Major input datasets colored blue. Major derived results colored orange. Intermediate transformation steps in gray.

These data were processed in several steps to identify and address data quality issues. The main steps of this process are illustrated in Fig. 6, and a detailed description is given in the following sections. The final result of the process is a dataset of areas and N harvests in 17 crop categories (Table 3), covering the entire time period defined for each of country. The resulting dataset is found in the data record^12^.

**Table 3:** The crop categories resulting from the crop data processing.

Data sources	Crop category	Number of source crop codes
FAOSTAT	Wheat	1
”	Barley	1
”	Grain maize	1
”	Other cereals	9
”	Oilseeds	15
”	Potatoes	1
”	Sugar beet	1
”	Pulses	9
”	Vegetables and other	41
”	Olives	1
”	Grapes	1
”	Other permanent crops	40
Eurostat and others	Green maize	1
”	Temporary grassland	1
”	Forage legumes	2
”	Fodder roots	1
”	Other forage crops	1

Some categories are based on data from a single crop code in the FAOSTAT or Eurostat crop production databases: these are major crops that together typically cover 50–80% of the cropland in each country. The full categorization is given in the data record^12^.

#### Rationale for crop categorization

The crop categories listed in Table 3 were chosen to produce a dataset that gives comprehensible and agronomically relevant information about major trends in crop mix and productivity. Specifically, the categorization was made based on the following considerations:

- The categories should contain crops with similar N yields and similar N yield changes over time. For example, wheat and grain maize have had a substantially steeper yield increase than other cereals over time and are therefore reported separately.
- The categories should be well-known categories of crops to simplify interpretation and comparison with other datasets. For example, although sugar beets and potatoes are comparable to cereals in terms of N yields and could possibly be grouped according to the previous criterion, we separated them since they are typically separated in agricultural statistics and models.
- The categories should make visible the characteristic differences in crop mix between countries. For example, we separated olives and grapes from other permanent crops since these two crops cover 20–30% of the cropland in four Mediterranean countries, compared to about 9% of the total European cropland.
- The number of categories should not be too large because with very small categories the signal-to-noise ratio declines, making any statistical analysis more difficult.

### Crop areas and harvests except fodder crops

From the FAOSTAT crop database, we extracted data on 121 arable and permanent crops. We converted the reported harvests to N quantities assuming crop N contents from Lassaletta *et al*.^17^. The same crop N contents are assumed for all countries in the whole period 1961–2019, even if it is likely that crop N contents have varied both geographically and over time, for several reasons. For example, N contents will tend to increase with dry weather and low yields; N contents will tend to increase with fertilizer rates; and N contents may be may be changed both up and down because of crop breeding. However, a more detailed analysis of these effects has not been possible in the scope of this study.

As described above, we then aggregated these crops to 12 categories listed in Table 3.

While the FAOSTAT database has a good level of coverage for most major crops (apart from fodder), there are sometimes data gaps where harvested quantities are reported without corresponding areas. This occurs mainly before 1985 in permanent crops (including olives) and to a lesser extent in the categories “Oilseeds” and “Vegetables and other”. For these crops, summing the individual crop areas in a country-year would sometimes result in considerable underestimation of the total harvested area. Similarly, summing areas and harvests separately for available crops would result in incompatible estimates of category areas and harvests.

In order to ensure consistent estimates of category areas and harvests, we therefore took the following approach.

For each crop category, we calculated the sums of available crop areas (*A*_sum_) and crop N harvests (*H*_sum_). In addition, based on the crops where both areas and harvests were available, we calculated category-level weighted average N yields (*Y*_est_). Since some area data were missing for individual crops, we additionally calculated an estimate of total category area *A*_est_ = *H*_sum_/*Y*_est_. When crop harvests are available but some crop areas are missing, the estimate *A*_est_ is equivalent to assuming that the weighted average N yield of crops with missing areas is equal to the weighted average yield of the other crops in the same category.

For each country and category, we then generated and inspected figures showing the time series of the category-level variables *A*_sum_, *A*_est_, *H*_sum_, and *Y*_est_ along with available crop-level data on area, harvests, and yields. If all crop categories had been present in all countries, there would have been 28 × 12 = 336 figures. However, some country × category combinations are absent (e.g., olive trees in Sweden) and in total there were 304 figures to inspect (available in the data record^12^).

Based on visual inspection of these figures, and in some cases cross-checking against other sources, we then chose on a case-by-case basis how to estimate the category area and harvest from available data. Note that we used available crop-level data to fill category-level data gaps. Filling all the data gaps on crop level have been very laborious and also unnecessary, since the aim was merely to make complete and consistent estimates on crop category level.

By default we used *H*_sum_ as estimate of the category harvest and *A*_est_ as estimate of the category area. We used these default estimates when they looked fairly smooth over time and no obvious and serious data gaps were present in the crop-level data.

However, sometimes there were data gaps in the crop-level data that motivated other adjustments on the category level. For example, one type of problem is when a category-level variable is completely missing. The most important example is that for olives in Greece, Portugal, and Spain, harvest data are available since 1961 but area data only since the 1980s. For olives in these three countries, we collected data based on national statistics^12,25–28^. In addition, we filled a small number of minor data gaps (e.g., harvests or areas completely missing during one or a few years) using constant extrapolation. A similar type of problem is when some crops within a category lack area and/or harvest data during a part of the period. This can create a false impression that the category’s N yield has suddenly changed abruptly. In a few such cases it appeared more plausible to extrapolate (or sometimes interpolate) areas or yields to obtain a dataset covering the whole period. Generally, these various adjustments made to the default estimates *H*_sum_ and *A*_est_ were rather small. The missing olive areas in Greece, Portugal, and Spain were by far the most important adjustments made in this process. In each country, there was less than one percent difference between the default estimate *H*_sum_ and the final adjusted estimate of total N harvests. Prior to 1985, these adjustments increased the total crop area by about 1.5% on average across all countries.

Finally, we also explicitly assigned zero harvests and areas where data were completely missing, to obtain a full dataset of the 12 FAOSTAT crop categories in each country.

### Fodder crop areas and harvests: overview

As mentioned above, the FAOSTAT crop production database excludes most fodder crops. Here, we instead assembled a fodder crop dataset using Eurostat crop statistics (see Table 1) and other sources.

Eurostat reports areas and harvests of arable and permanent crops in a hierarchy of crop codes^23,29^. The most important category of fodder crops in this hierarchy is “Plants harvested green from arable land” (crop code G0000), which is further subdivided in a number of subcrops as shown in Table 4. In addition, we included the Eurostat category “Other root crops n.e.c.” (R9000) which mainly accounts for fodder roots, for example *Beta vulgaris* (known by many names, including fodder or forage beet, or mangold, mangelwurzel, etc.) and several *Brassica* species (rutabaga/swede, turnip, etc.)^23^. Crop code R9000 does not include roots for seed or human consumption. Other crops used completely or partly for animal feed, including grain legumes, cereals harvested for grain, sugar beets, potatoes, etc., are accounted for in the FAOSTAT database^30^. To our knowledge, the crop codes G0000 and R9000 together account for all the major European fodder crops not included in the FAOSTAT database.

**Table 4:** Excerpt from Eurostat’s hierarchy of crop codes^23^.

Crop code	Label
G0000	Plants harvested green from arable land
**G1000**	**Temporary grasses and grazings**
G2000	Leguminous plants harvested green
**G2100**	**Lucerne**
**G2900**	**Other leguminous plants harvested green n.e.c**.
**G3000**	**Green maize**
**G9000**	**Other plants harvested green from arable land**
G9100	Other cereals harvested green (excluding green maize)
G9900	Other plants harvested green from arable land n.e.c.
R0000	Root crops
R1000	Potatoes (including seed potatoes)
R2000	Sugar beet (excluding seed)
**R9000**	**Other root crops n.e.c**.

The crops included in this study and referred to as “fodder crops” are marked in bold face.

In the following sections we describe in detail how we combined data from Eurostat with other sources corresponding to the Eurostat crop codes G1000, G2100, G2900, G3000, G9000, and R9000. The results are available in the data record^12^.

### Fodder crop areas

#### Data extraction from the Eurostat ACS database

Harvested areas are reported in Eurostat’s ACS database^23^. Data coverage in the Eurostat ACS data varies widely. For some countries, especially the early members of EEC and EU, the area data are complete back to the 1950s. For the more recent EU member states, the data coverage typically starts around the time of their accession application to the EU. For Croatia, Estonia, Latvia, Lithuania, and Slovenia, which in this study are covered starting in 1992, the data coverage is fairly complete. However, for the former communist states of Bulgaria, Czechoslovakia, East Germany, Hungary, Poland, and Romania, which in this study are covered starting in 1961, there are no data prior to 1987 in the Eurostat database. Eurostat also lacks data during some periods for several countries in western Europe. In a few cases we cross-checked suspected errors and gap-filled area data from the Eurostat Farm Structure Survey (FSS)^29^ (see Table 1). However, the FSS data generally have smaller coverage than the ACS and are not entirely comparable in scope and methods, so we used it very sparingly.

Some special treatment was needed for crops G9000 and R9000. Crop code G9000 is not reported in the Eurostat ACS, but we summed it from available data of G9100 and G9900. The reason to merge these two crop codes is that their reported areas often fluctuate in such a way to suggest that the same crops have been reported variably as G9100 or G9900; thus, data gaps for the combined G9000 area are fewer and easier to fill than for the individual G9100 and G9900 areas. For R9000 (fodder roots), data were almost never explicitly stated, but could in many cases be calculated as R0000–R1000–R2000^23^.

#### Gap-filling and other adjustments to the Eurostat fodder crop areas

In most countries, the Eurostat data on fodder areas are fairly smooth, complete, and internally consistent since around year 2000. Before this period, several countries have data gaps and/or report large, sudden changes which we intepreted as potential errors. A reason to expect some errors and inconsistencies is that the nomenclature used in older national statistics likely is incompatible with the current Eurostat crop nomenclature, which may cause problems in the translation of old data to the Eurostat database. However, since considerable shifts in fodder crop areas actually have occurred since 1961 in most European countries, it is not always straightforward to determine whether abrupt changes in reported areas are reporting errors or accurate representations of historical developments. We therefore scrutinized and cross-checked the Eurostat data against other sources, filling data gaps and making other adjustments to reconcile major discrepancies. The collected dataset on fodder crop areas, as well as figures showing the stepwise gap-filling of fodder crop areas, are available in the data record^12^.

Fodder roots were important crops in the first half of the 20th century in several European countries, but areas then declined as they were replaced by other, less labor-intensive fodder crops^31^. Therefore we paid special attention to filling data gaps in R9000 areas during the 1960s–1970s.

Before listing the data sources and adjustments country by country, we specifically mention the common approach used to fill the long 1961–1986 data gaps in Bulgaria, Czechoslovakia, East Germany, Hungary, Poland, and Romania. We mainly used data from reports of the Economic Research Service of the US Department of Agriculture (USDA ERS), which during the 1960s–1980s collated information from the statistical yearbooks of the socialist states in a series of reports^32–36^. These reports cover the years 1960 and 1965–1987, and give areas for three categories of fodder crops: “feed roots”, “corn silage”, and “hay”. We assigned the former two crop codes R9000 and G3000, which in the overlapping year 1987 agreed perfectly with the Eurostat ACS. The last category, “hay”, is more complicated: it may refer to a mix of annual and perennial crops harvested green, predominantly forage legumes in pure stands or mixed with grass, cereals and cereal/legume mixtures, and possibly pure grass cultivation on arable land. The USDA ERS “hay” category clearly excludes harvests from permanent grassland. Since there is usually one year of data overlap between the USDA ERS statistics and the Eurostat ACS in 1987 for these countries, we could usually conclude that the “hay” area then corresponded to combination of crop codes G2100, G2900, and sometimes G9000. Country by country, we decided on a combination of Eurostat crop codes to match against the “hay” area, and then divided the 1960–1986 “hay” area between them in proportion to their their 1987 areas. Temporary grasslands (G1000) account only for a few percent of the fodder crops in most of Eastern Europe, and we therefore mostly extrapolated the earliest available G1000 areas back to 1961. Country-specific details are elaborated below.

The remainder of this section lists the data sources and adjustments country by country. For brevity, we omit some descriptions of the following minor adjustments: interpolation of minor data gaps, sometimes using data from 1960 or 2020; extrapolation of minor fodder crops accounting for a small share of the total fodder area; removal of obvious outliers.

**Austria**. The Eurostat ACS data are complete and consistent since the start in 1980. Data for 1960 and 1970–79 were filled using national statistics and data from the FAO 1960 World Census of Agriculture^37–39^. Remaining data gaps interpolated and extrapolated.

**Belgium and Luxembourg**. The Eurostat ACS data are almost perfectly complete and consistent since the start in 1955. Minor data gaps in G2100 and G2900 areas interpolated.

**Bulgaria**. The Eurostat ACS data are almost complete and consistent since the start in 1987. In 1960–1986, we used G3000 and R9000 areas from USDA ERS publications^34–36^ as explained above. In 1987, Eurostat’s combined area of G2100 and G2900 matches the USDA ERS “hay” area, so we divided the 1960–1986 hay area between these crop codes in proportion to their 1987 shares, and extrapolated G9000 and G1000 values constant to 1961.

**Croatia**. The Eurostat ACS data are complete from the start in 2000. We extrapolated the areas back to 1992.

**Czechia**. The Eurostat ACS data are complete from the start in 1987 apart from G1000 which is reported at around 10% of the fodder area since 2011. A lone G1000 value in 1999 is conspicously close to the temporary 1995–1999 decrease in G2900, suggesting a temporary classification change in temporary grassland and legume-dominated crops. Since G1000 data are largely missing, we chose to discard the 1995–1999 decrease in G2900 area and interpolate surrounding values while extrapolating the 2011 G1000 area constant back in time to 1987.

**Slovakia**. The Eurostat ACS data are complete and consistent from the start in 1987 except a minor gap in G2100 and G2900 areas which we interpolated.

**Czechoslovakia**. Areas for 1987–1992 were taken as the sum of the adjusted Eurostat ACS data for Czechia and Slovakia. In 1987, Eurostat’s combined area of G2100, G2900, and G9000 matches the USDA ERS “hay” area, so we divided the 1960–1986 hay area between these crop codes in proportion to their 1987 shares. G1000 was extrapolated constant to 1961.

**Denmark**. The Eurostat ACS data are fairly complete and consistent back to 1955, except for the G9000 area which fluctuates considerably. A closer inspection shows that 1973–2009, G9900 has a large area share while G9100 is not reported; and from 2010 the G9900 area is zero while G9100 has a smaller share. Before 1973, the Eurostat database does not report G9100 or G9900 areas. National statistics show that this apparent discontinuity arises because the reported G9900 area for some years, in addition to cereals harvested green, also includes aftermath, i.e. late season harvests or grazing after other crops. The aftermath at its peak in year 2000 accounted for about half the reported fodder area on arable land but less than 10% of the harvested feed value^40,41^. Considering the incomplete data coverage and the minor importance of the aftermath in terms of harvested quantities, we replaced the Eurostat G9000 area by a complete record of cereals harvested green (i.e., corresponding to G9100) based on national statistics^42,43^. The national statistics prior to 1982 report the combined area corresponding to G3000 + G9100, so to avoid double counting we subtracted the G3000 area as reported by Eurostat.

**Estonia**. The Eurostat ACS data are mostly complete from 1991. A data gap in the G1000 area was filled by difference since the G2000 data clearly includes the later G1000 area until 2003. We also filled minor data gaps in G2100 and G2900 using national statistics^44^.

**Finland**. The Eurostat ACS data are complete since 1998. We used national statistics^45^ to fill the G1000 area, which in 1998 covered more than 95% of the fodder area. Fodder roots made a minor contribution in Finland even in the 1950s and 1960s^46^ when they were much more common in other countries. The main feed root seemingly was potatoes^46,47^, which is already accounted for in the FAOSTAT crop database^30^. Considering the lack of further data and the dominance of G1000 in the fodder production we extrapolated the 1998 area of other fodder crops back to 1961.

**France**. The Eurostat ACS data are complete since 1961 apart from a few minor data gaps which we interpolated.

**Germany**. The Eurostat ACS data cover all the fodder crops starting in 1955, but geographically covers only West Germany until 1989. To complete the period 1961–1989, we estimated East Germany’s fodder crop areas in 1989 data from Eurostat ACS and USDA ERS^34–36^ data as follows. We estimated East Germany’s fodder crop areas in 1989 as the Eurostat increment in fodder crop areas 1989–1990, an estimate which builds on the assumption that both West and East German fodder crop areas were approximately constant 1989–1990. East Germany’s 1960–1987 areas of G3000 and R9000 were then taken from the USDA ERS data. The estimated 1989 area of G1000, G2100, G2900, and G9000 matched the USDA ERS 1987 “hay” area, so we divided the 1960–1987 hay area between these crop codes in proportion to their 1989 shares. The remaining minor data gaps were interpolated.

**Greece**. While the Eurostat ACS data appear complete and internally consistent since year 2000, they are difficult to reconcile with older Eurostat data and data from other sources. For the period 1969–1986, the Eurostat ACS data suggest that alfalfa (G2100) is the dominating arable fodder crop varying around 150–200 thousand hectares (kha). Similar alfalfa areas are reported from the 1950s until 2017 in multiple overviews of Greek fodder production as well as recent national statistical publications^48–53^. In the Eurostat database, however, there are almost no fodder crop data reported for 1988–1999, and from year 2000 the alfalfa area is reported around 10–15 kha. For other forage legumes, the Eurostat data consistently report zero area, while several sources report fairly constant areas in the range 35–70 kha^48,50–53^. The area data for fodder crops in the Eurostat FSS 1990–2016^29^ are incomplete and offer few additional insights. Based on these considerations, we made the following adjustments to the Eurostat data: discarded the Eurostat area data for G2100 starting in year 2000 and extrapolated the fairly constant areas 1969–1986 to the whole period 1961–2019; assumed a constant G2900 area of 50 kha; interpolated the G9000 area data which appear broadly consistent with various data sources; and extrapolated the average G1000 area from year 2000 back to 1961.

**Hungary**. The Eurostat ACS data on Hungary’s main fodder crops, forage legumes and green maize, are mostly complete from 1987. The USDA ERS data^34–36^ provide areas of G3000 and R9000 back to 1960. The G9000 area is missing 1987–1997. Since the USDA ERS “hay” area exceeds the combined G2100 and G2900 area in 1987, we estimated the G9000 area as the remainder of the hay area in 1987, and then divided the 1960–1986 hay area between G2100, G2900, and G9000 in proportion to their 1987 shares. The resulting G2100 area estimate in 1961–1987 agrees remarkably well with data from the Hungarian Central Statistical Office^54^. For G1000, the few available data indicate that the area is very small compared to the total fodder area, and we extrapolated the 2003 area constant back to 1961.

**Ireland**. Temporary grassland (G1000) clearly dominates fodder production on cropland. However, a considerable area of temporary grassland has been reclassified to permanent grassland around 1996–2016 although sources disagree on the exact size and timing of this shift. The Eurostat ACS data reports a drop from about 0.7 Mha to 0.1 Mha between 1996 and 2000, and a corresponding increase in permanent grassland (crop code J0000) between 2001 and 2002. The Eurostat FSS reports a similar drop in G1000 area between 2013 and 2016. The FAOSTAT database reports a simultaneous change in both temporary and permanent grassland between 2006 and 2008. For consistency with the FAOSTAT permanent grassland area (details below) we used FAOSTAT’s temporary grassland area data during 2000–2006, the only period where it disagrees with the Eurostat ACS. For the remaining fodder crops, area data are almost complete from the start in 1955.

**Italy**. The Eurostat ACS data are mostly complete for G2100, G2900, G3000, and R9000 since 1970. We filled a 1989–2013 data gap in G2900 by interpolation. For G1000 and G9000, the Eurostat annual crop statistics are incomplete and somewhat erratic. In the years where data for both G1000 and G9000 are available, their sum shows a smooth decline from around 1.2 Mha in the 1980s to about 0.8 Mha in 2014, suggesting that crop classifications may have changed more than actual areas. National statistics from 2006–2018^55^ support this interpretation, although the national statistics cannot easily be matched to the Eurostat crop codes. A detailed expert overview from 1977^56^ agrees well with the G1000 and G9000 data for the 1970s. Considering all this, we filled the G1000 and G9000 data gaps by interpolating their area sum between 1986 and 2014 and dividing the resulting total in proportion to their 1986 areas. Remaining data gaps were filled by extrapolation from the 1970s back to 1961.

**Latvia**. The Eurostat ACS data are mostly complete for G3000, G9000, and R9000 since 1987. Areas of G1000 are reported since year 2000, completely dominating the fodder area. In 1987–1999, reported areas for G2000 appear to include what is later reported as G1000, suggesting a change in crop classifications. We extrapolated the small areas of G2100 and G2900 reported since 2015 constant back to 1992 and assigned the remainder of the 1992–1999 G2000 area to G1000.

**Lithuania**. Like in Latvia, the Eurostat ACS data reports no G1000 area prior to 2000, but a G2000 area which likely includes the later G1000 area. We extrapolated the 2001 areas of G2100 and G2900 back to 1992 and assigned the remainder of the 1992–1999 G2000 area to G1000. This leads to an estimated 95% decrease in the G1000 area between 1999 and 2001, which agrees with an increase in Lithuania’s permanent grassland area registered in the Eurostat and FAOSTAT databases since 2001. Most likely this accurately reflects a heavy decrease in the resowing of grasslands in Lithuania which occured after the collapse of the Soviet Union^57^. We extrapolated the G1000 area from 1999 to 2000 to agree with the timing of this change in the reported permanent grassland areas.

**Netherlands**. The Eurostat ACS data are complete since 1955 except for minor gaps which we interpolated. The data show an abrupt increase in temporary grassland (G1000) area from less than 40 kha in the mid-1990s to about 200 kha in the mid-2000s, a change which is also reflected in decreased permanent grassland areas in the Eurostat and FAOSTAT databases.

**Poland**. The Eurostat ACS data are mostly complete since the start in 1987, but only partly consistent with other data sources. The USDA ERS data for 1960–1987^34–36^ precisely match the G3000 and R9000 areas in 1987 and we therefore used these without modification. However, the USDA ERS “hay” area in 1987 is considerably smaller than the combined Eurostat G2100 and G2900 areas, which suggests a reporting error in at least one of the datasets. Several factors strongly suggest that the Eurostat G2100 and G2900 areas are both incorrect in 1987–2001. First, Eurostat’s reported G2900 area falls by more than 1.5 Mha in 1987–1998, a change rate which appears unlikely even given the rapid changes taking place in Poland starting in the late 1980s. Second, national statistics from Polish statistical yearbooks^58–61^ report that the area of fodder legumes has never been as high as 1.5 Mha in Poland, and specifically suggest a mistake in crop code assignments since Eurostat’s G2100 areas from 1987 to 2001 exactly equal the total areas of perennial legumes according to national statistics. Third, area data from the Eurostat FSS in 2003 also suggest that Eurostat ACS data for G2100 and G2900 are incorrect until year 2001. Based on this, we discarded the Eurostat ACS areas of G2100, G2900, and G9000 in 1987–2001.

The USDA ERS “hay” area varies between 1.4 and 1.8 Mha in 1960–1987. The Polish statistical yearbooks do not give sufficient information to divide this between crop codes G1000, G2100, G2900, and G9000. However, data from the FAO 1960 World Census of Agriculture^39^ shows that clover was the main forage legume (around 0.6 Mha). Alfalfa covered about 0.13 Mha, about 13% of the pure forage legume area. Throughout the 1960s–1980s, the statistical yearbooks show that perennial legumes covered about half the hay area^58,59^. Similarly, an expert summary of national statistics in 1965 loosely described the fodder area except maize and fodder roots as consisting of 48% pure forage legumes and 52% of clover/grass and pure grass^62^. In line with this, Eurostat’s G1000 area for 1987 corresponds to 29% of the 1987 hay area. Based on these data, we divided the USDA ERS hay area in 1960–1987 using the following fixed proportions: 29% G1000, 6% G2100, 42% G2900, and the remaining 23% G9000. We interpolated the remaining data gaps.

**Portugal**. The Eurostat ACS data are incomplete. The total G0000 area is reported roughly constant since 1978. An almost constant share of around 9% G1000 and 18% G3000 is reported since 1991. The major area appears to be G9000, but its area is only reported since 2011. Quantitative data from other sources appear to be scarce^63^. A paper^64^ from 1990 describes cereal/legume mixtures (i.e., G9000) as the main arable fodder, which agrees with recent Eurostat data. Considering the near-constant G0000 area since 1978 and the near-constant shares of different fodder crops, and that few additional data could be found, we extrapolated available data constant back to 1961.

**Romania**. The Eurostat ACS data are complete and consistent since the start in 1987. Areas of G3000 and R9000 1960–1987 were filled from USDA ERS publications^34–36^ as explained above. In 1987, Eurostat’s combined area of G2100, G2900, and G9000 precisely matches the USDA ERS “hay” area, so we divided the 1960–1986 hay area between these crop codes in proportion to their 1987 shares, and extrapolated the very small G1000 area constant from 2005 back to 1961.

**Slovenia**. The Eurostat ACS data are almost complete from the start in 1991. Some minor data gaps were filled using national statistics^65^.

**Spain**. The Eurostat ACS data are almost complete since 1965. We extrapolated the 1965–66 areas back to 1961.

**Sweden**. The Eurostat ACS data are fairly complete from the start in 1992. We filled the data gaps back to 1961 using national statistics^66^.

**United Kingdom**. The Eurostat ACS data are almost complete since the start in 1955. The main exception is fodder roots (R9000), for which Eurostat data are available since 2000. We completed the record using data from national surveys^67–69^ covering all major fodder roots: turnips, swedes, and fodder beets (including mangolds, which are distinct from fodder beets in British terminology^31^). For G9000, a minor issue is that the 2010–2011 areas are reported identical to G3000 areas, likely by mistake. We discarded these data and filled by interpolation. We also extrapolated G9000 constant from 1970 back to 1961.

### Fodder crop harvests

We used Eurostat ACS data to estimate fodder crop N harvests. The ACS production data have two important limitations: (1) they are incomplete, even more so than the area data, and (2) they have several inconsistencies related to the water content of the harvest (details below). For these reasons, it was not possible to establish time series of fodder crop yields for more than a few crop/country combinations. In the few cases where long-term time series are available, they however show that fodder crop yields on average have increased relatively slowly.

Considering the lack of data, and that the period 2000–2019 has the best data coverage, we decided to estimate country/crop specific yields in 2010 from available data. Between 1961 and 2019, we assumed based on long-term statistics from Austria, France, Hungary, Italy, Poland, and Sweden^28,37,56,58,59,70–74^ a linear increase such that the 1961 yields are 75% of the 2010 yields. The following subsections describe the estimation of 2010 yields from available data.

#### Estimating the dry matter yields of fodder crops

The nominal water content of the harvest data is a central concern since harvests may be reported with different nominal water content. Some countries report in dry matter (0% water), while others use crop-specific nominal water content typically between 12% (hay) and 65–80% (e.g., green maize and other silage crops).

The Eurostat ACS data since year 2000 accounts for these differences by reporting the water content (“humidity” in Eurostat’s nomenclature) along with the harvested quantities^28^. There are two different datasets, one in national humidity (0–88% water content) and one in EU standard humidity (always 65% water content for plants harvested green)^28^. The coverage of humidity values (since year 2000) is not complete, which means that sometimes the harvest is only given in national humidity basis. Prior to year 2000, all the data are given in national humidity basis, without corresponding humidity values.

To construct a harmonized dataset of dry matter yields for each country/crop combination, we studied and compared four time series of available yield values: (1) in national humidity, (2) in EU standard humidity, (3) in dry matter based on the national humidity dataset, and (4) in dry matter based on standard EU humidity dataset. For crop code G9000, we calculated production-weighted average yields from data on crop codes G9100 and G9900. Based on a close inspection of these data and sometimes cross-checking against national data sources, we identified the following types of possible reporting errors:

- Some crop yields are reported equal in national and EU humidity, although the corresponding humidity values differ. This creates two different dry matter yields, at most one of which could be accurate. This possibly reflects a mistake in the conversion between national and EU humidity. In these cases we typically used the national data.
- Sometimes, the reported yield of a crop changes drastically from one year to the next such that the older yield in national or EU humidity roughly equals the new yield in dry matter, or vice versa. In these cases, one of the two yield levels could typically be ruled out as implausible.
- Some calculated dry matter yields seem implausibly low. In some of these cases it could be deduced that a yield reported with a nominal water content of 65% was actually in dry matter or hay basis (i.e., about 85% dry matter).
- Some yield values seem implausibly high or low compared to neighboring years or similar countries without any apparent reason.

Based on these considerations, we selected a subset of yield values for each country/crop, which we then averaged to an estimate of the 2010 (reference year) yield. We aimed to use 2000–2019 data if possible, not only because they best represent the 2010 yields but also because the more recent data are more complete and consistent. We used data from the 1990s in a few cases where no 2000–2019 were available. We used dry matter yields with only a few exceptions: for the crops G1000, G2100, and G2900, we sometimes used yields in national humidity basis if these appeared to be reported as hay or dry matter, assuming a dry matter content of 85%; and for fodder roots (R9000), humidity values are not reported and we uniformly assumed a dry matter content of 16%^75^. Figures illustrating data selection are available in the data record^12^.

#### Accounting for grazing on cropland

We considered the complication that cropland, especially temporary grasslands (G1000), to some extent is grazed in addition to the mechanical harvest. The harvest statistics for temporary grassland appear to account only for mowing which means that they underestimate the total crop production. Mixed mowing and grazing appears to occur in temporary grasslands throughout Europe^76,77^, but quantitatively it is probably most important in the Nordic countries where temporary grassland occupies a considerable share of the cropland and is grazed fairly commonly.

In fact, grazing may also occur in several of the fodder crops as well as in other crops, between or after harvests. However, we consider temporary grasslands as the probable main source of grazing intake on cropland, and considering the lack of data on this topic we make an estimate of grazing intake on cropland accounting only for temporary grassland.

Relevant data to accurately estimate the grazing component of temporary grassland production are very scarce, but a recent investigation of Swedish data shows that grazing contributes about 20% in addition to the mechanical harvest of temporary grassland^78^. At least in Finland and Sweden, similar proportions of temporary grassland are used exclusively for grazing^45,79^. Considering that no further information could be found, we inflated the G1000 yield estimates by 20% in all the countries.

#### Filling of remaining data gaps in fodder crop yields

A few remaining data gaps were filled by extrapolating crop yields from neighboring countries with similar climate and agricultural productivity. In some cases we averaged yield values from multiple neighboring countries. The following data were extrapolated from other countries:

- Belgium (G9000): average of Luxembourg and the Netherlands
- Czechoslovakia (all fodder crops): average of Czechia and Slovakia
- Germany (G9000): from France
- Greece (all fodder crops): from Bulgaria
- Ireland (G1000, G2900, G9000): averages of Belgium, France, Germany, and the Netherlands
- Italy (G2100, G9000): average of France and Austria
- Latvia: (G2100, G2900): from Lithuania
- Luxembourg (R9000): average of France and the Netherlands
- Portugal (G1000, G9000, R9000): from Spain
- Sweden (R9000): average of Latvia and Lithuania
- United Kingdom (G1000, G2100, G9000, R9000): averages of Belgium, France, Germany, and the Netherlands

#### Estimation of fodder crop harvests

We finally averaged the selected yield values for each country/crop, thus producing the estimates of 2010 dry matter yields. From these, we estimated N yields assuming N contents listed in Table 4.

Finally, we calculated the N harvest for each of the fodder crops in each country by multiplying the estimated yield time series by the gap-filled area time series.

#### Aggregation of fodder areas and harvests to categories

The six Eurostat fodder crops (Table 4) were ultimately aggregated to five categories following the considerations described above. Specifically, we aggregated alfalfa (G2100) and other forage legumes (G2900) into one category.

### Cropland and grassland areas

Several of the results calculated in later sections depend on the total cropland area as well as on the areas of permanent and temporary grassland. This section explains how we estimated these areas from available data sources.

As an estimate of cropland area in use, Lassaletta *et al*.^17^ used the sum of harvested crop areas, except when this sum exceeded the total cropland area reported in the FAOSTAT database, in which case the FAOSTAT cropland area was used instead. The sum of harvested crop areas can exceed the FAOSTAT cropland area due to multicropping or intercropping. We followed the same approach as Lassaletta *et al*., with the only difference that we used the sum of adjusted crop categories (henceforth called the crop area sum) rather than the sum of individual FAOSTAT crops. Some examples of the crop area sum compared to the FAOSTAT cropland are shown in Fig. 2.

Permanent grassland areas, following Lassaletta *et al*.^17^, were taken from the FAOSTAT database (“Land under perm. meadows and pastures” in the land use dataset). These areas were used in estimates of the allocation of synthetic and manure N inputs to cropland (details below).

The FAOSTAT land use data at the time of our study was only available until 2018. We considered two options for filling the data gap in 2019. Eurostat data on permanent grassland and cropland could be used, or the FAOSTAT data for 2018 could be extrapolated constant to 2019. In general, the two methods would produce very similar results. However, since the FAOSTAT and Eurostat data series on permanent grasslands in a few countries differ substantially, we chose to extrapolate the FAOSTAT land use data constant from 2018 to 2019 as it probably gives the most internally consistent results.

Temporary grassland areas were taken equal to the gap-filled crop category Temporary grassland (see Table 3).

#### Assessing the accuracy of the estimates of cropland in use

Our approach may lead to an overestimate or an underestimate of actual cropland area in use. The crop area sum may overestimate the cropland in use if some areas are counted more than once due to multicropping or intercropping. This is the reason to set the FAOSTAT cropland area as an upper limit to cropland in use. The reported cropland area may also overestimate the cropland in use since it may include considerable areas of fallow land.

In principle, a more direct estimate of cropland in use would be the reported cropland area minus fallow land, but this is not an option since available data on fallow land areas in the FAOSTAT and Eurostat databases are much too incomplete to cover the whole 1961–2019 period.

Instead, to test the accuracy of our estimates, we compared the crop area sum to cropland minus fallow land using data from the Eurostat database which has the most complete records of fallow land. The most common result of this test is that the areas match within a few percent (less than ± 5% difference in 75% of 971 country-years with data on fallow area). See figures in the data record^12^ for details.

We also compared the crop area sum to the FAOSTAT cropland area. The crop area sum exceeds the FAOSTAT cropland by at least 1% in 163 country-years, about 12% of all 1308 country-years. The only major exceedances, e.g., more than 10% exceedance for at least 3 years, are in the 1960s and 1970s in Italy, Portugal, and Romania. See figures in the data record^12^ for details. At least in Romania most of the difference is explained by inter- and multicropping of cereals with beans and squash or pumpkins which was a common but gradually decreasing practice during the 1960s and 1970s^80,81^. In general, however, we have not been able to systematically determine the extent of inter- and multicropping.

In summary, these results show that the crop area sum is usually a good approximation of cropland in use in Europe, especially after the 1970s.

### Symbiotic N fixation

We estimated symbiotic N fixation in pulses and forage legumes using the same method as Lassaletta *et al*.^17^, i.e., assuming a linear relationship between the fixed N and the harvested N yield *Y*,

1 $${\rm{BNF}}=Y\cdot {\rm{Ndfa}}\cdot \frac{{\rm{BGN}}}{{\rm{NHI}}},$$

where Ndfa is the share of plant N derived from the atmosphere, BGN is the ratio between total and above-ground plant N, and NHI is the N harvest index, i.e., the ratio between harvested and total above-ground plant N. We used the same crop-specific parameter values as Lassaletta *et al*.^17,82^.

For crop code G1000 (temporary grassland, including grass/clover mixtures) we assumed that 25% of the dry matter harvest was forage legumes;^5,17^ for G2900 (pure forage legumes, sometimes mixed with grass) 90% forage legumes; and for G9000 (a variety of crops, including cereal/legume mixtures) 25% forage legumes (see also Table 5, and Eurostat’s ACS handbook^23^).

**Table 5:** N contents assumed for the fodder crops.

Crop code	N content (% N of DM)	Comment	References
G1000	2.3	75% grass (2.0% N), 25% clover (3.3% N)	^182–185^
G2100	3.0		^186^
G2900	3.2	90% clover (3.3% N), 10% grass (2.0% N)	^182–185^
G3000	1.2		^187,188^
G9000	2.0	75% cereal forage (1.6% N), 25% legumes (3.0% N)	^189–191^
R9000	1.3	Fodder beet	^75^

The resulting parameter values are available in the data record^12^.

### Atmospheric N deposition

Lassaletta *et al*.^17^ developed country-specific time series of N deposition rates in 1961–2013. We used these, extrapolated constant in the period 2014–2019, to estimate atmospheric N deposition input to cropland by multiplying by the estimated cropland area in use.

### Synthetic N fertilizer consumption

Time series of agricultural synthetic N fertilizer consumption in European countries are available in several international databases:

- FAOSTAT has a dataset representing agricultural use of fertilizers, based primarily on data reported by countries. FAOSTAT fills data gaps using various imputation methods, e.g., based on trade and production data^83^.
- Eurostat has a dataset representing agricultural use of fertilizers, based on data reported by countries^84^.
- Eurostat additionally publishes a dataset representing total national fertilizer sales, using data from Fertilizers Europe^85^.
- The International Fertilizer Association (IFA) publishes a dataset representing national consumption. It is based on sales data from the fertilizer industry, and gap-filled using production and trade data and N budgeting^86^.

In principle, national sales of fertilizers is not equal to agricultural use because (1) stock changes between years can shift agricultural use compared to sales, and (2) sales also cover non-agricultural uses such as parks and lawns. However, due to lack of data, the international datasets of agricultural use do not systematically account for stock changes and non-agricultural use^83,84^.

There is no immediately apparent way to judge which of these four datasets best represents the actual history of agricultural use. We therefore inspected and compared the four datasets to assess (1) to what extent they cover the studied time period, (2) whether the data seem consistent and plausible, and (3) how well the datasets agree with each other. The four datasets sometimes disagree considerably. By far, the longest and most complete datasets are provided by FAOSTAT and IFA. While these two datasets are mostly smooth and consistent, in a few exceptional cases they exhibit implausible jumps in consumption (e.g., by 50% or more) from one year to another. The FAOSTAT database has somewhat more such episodes, and moreover disagrees with the other three datasets more often than the others. While agreement between several datasets is no guarantee for their correctness, it seems likely that the majority vote of these partly independent estimates is the best estimate. The IFA dataset generally agrees well with the Fertilizers Europe dataset. The Fertilizers Europe dataset arguably exhibits the fewest implausible jumps but covers a shorter time period.

Based on these results, we chose to use the Fertilizers Europe dataset^85^ where possible, and in second place the IFA dataset^86^. These datasets together (1) have almost complete coverage of the country-years in this study, (2) mostly appear consistent and plausible, and (3) usually agree well with a majority vote of the four datasets. We used FAOSTAT data for Finland 1961–1984 and Slovenia 1992–2006 because the IFA data appeared implausible. In addition, a small number of data gaps were filled using FAOSTAT data (Croatia 1992–1993, Czechia and Slovakia 1993) and Eurostat country consumption data (Belgium and Luxembourg 2000–2019).

Figures showing the comparison of the four datasets and the selected data are available in the data record^12^.

### Share of synthetic N fertilizer to cropland

**Fig. 7:**
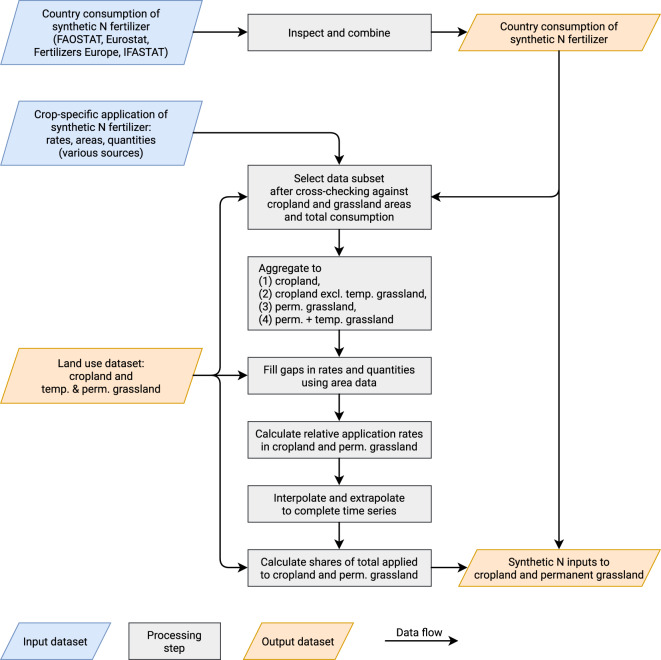
Illustration of main data sources and transformation steps used to estimate the application of synthetic fertilizer on cropland and permanent grassland. Major input datasets colored blue. Major derived results colored orange. Intermediate transformation steps in gray.

Following Lassaletta *et al*.^17^ we calculated the synthetic N fertilizer input to cropland by multiplying the total consumption by country-specific time series of the shares applied to cropland. Although data on these shares are scarce, it is well known that several European countries have long histories of large synthetic N inputs to permanent grassland. In this paper, we present a revised estimate of these shares, following same approach as Lassaletta *et al*., but adding a significant amount of additional data for several countries. To make sense of the incomplete and sometimes contradictory data we used several techniques to interpret and gap-fill the data on a country-by-country basis. Figure 7 gives an overview of the steps taken. In the following subsections we first describe the overall process in more detail and then provide country-specific details.

Ideally, the inputs of synthetic N fertilizer would be further disaggregated, e.g., to the 17 crop categories for which areas and harvests are reported in this study (Table 3). However, there are no datasets that enable comprehensive and reliable estimates of synthetic N fertilizer inputs on the level of individual crops or crop categories for the time period and countries covered in this study. As will be detailed below, pan-European crop-and-country-specific data on fertilizer inputs are available only for a few years between the 1990s and today. Further research on this topic would be valuable, and could use, for example, a combination of mass balances, statistical surveys, expert estimates, and crop-specific fertilizer recommendations from throughout the decades in different countries to establish plausible estimates of how synthetic N fertilizers have been allocated between crops. Such an exercise, however, could prove rather labor-intensive and moreover would necessarily imply a level of uncertainty which we have decided is not acceptable in the scope of this study.

Figures illustrating the final results are available in the data record^12^.

#### Data collection and interpretation

Three main categories of data were used. First, a few countries (France, Ireland, the Netherlands, and the United Kingdom) have carried out repeated national statistical surveys on grassland and cropland fertilization. Although each of these datasets have their idiosyncracies and required additional data processing (see below), we used them where possible since they are the most consistent and complete datasets available. Second, crop-specific fertilizer datasets from the fertilizer industry have been published in collaboration with FAO during the 1990s and early 2000s^87–89^. In addition, a similar unpublished dataset from EFMA^90^ (now Fertilizers Europe) gives crop-specific fertilizer rates for the crop year 2005/2006. These datasets are extremely valuable since they cover all the countries in this study during at least one year. The crop-specific datasets were later aggregated and further processed as explained in the following paragraphs. Third and last, we collected a range of expert estimates and other data from various literature sources. This last category is the least dependable type of data, and we used it only after exhausting other possibilities. However, given the general and long-running scarcity of data on grassland fertilization^91^, there are in many cases no alternatives to expert estimates prior to the 1990s. For all these data sources, as far as possible, we followed the references to the original data source and collected the data from there. In each case the citations in this paper point to the publication from which we collected the data.

All the data were collected in a table with columns for fertilizer rate *R*, fertilizer quantity *Q*, and crop area *A*^12^. Sometimes only one or two of these numbers were available. Each row in the table contains data from one publication concerning one country-year-crop combination.

We classified each row as belonging to one of the land categories cropland (C), permanent grassland (PG), temporary grassland (TG), non-grass cropland (C–TG), total grassland (PG + TG), or in some cases only the fertilized portion of grasslands (denoted PGf, TGf, or PGf + TGf). The main point of the land categories is that they enable the calculation of the share applied to cropland, *Q*_C_/(*Q*_C_ + *Q*_PG_). However, they also helped with the intermediate tasks of interpretation and consistency checking, aggregation, and gap-filling:

- Consistency checking and interpretation. A significant complication in using the FAO/EFMA datasets^87–90^ is that the crop categorization for some countries includes temporary grassland under the “grassland” item and for other countries under another item, usually “fodder (other)”. Since the final goal was to separate permanent grassland from cropland, it was necessary to work out what each “grassland” item refers to. This issue could usually be resolved since these datasets also report crop areas corresponding to the different fertilizer rates. To assign the appropriate land category to such items, we cross-checked the fertilized areas according to the FAO/EFMA datasets against the areas according to FAOSTAT/Eurostat, and thus could in most cases unambigously determine whether the “grassland” fertilizer rates referred to permanent (PG) or total grassland (PG + TG). We used the FAO/EFMA fertilizer data only when the summed fertilizer quantities and areas matched the previously estimated country-level fertilizer quantities and land category areas.
- Aggregation. Several data sources, including national statistical surveys and the FAO/EFMA datasets, report data for individual crops. In these cases, we assigned land category labels (C, PG, C−TG, etc.) to the individual crops that together constitute such a land category. We then calculated, e.g., the average application rate on cropland *R*_C_ as an area-weighted average of the individual crop rates. Where possible, we assigned labels C and PG since this allows the direct calculation of the final result *Q*_C_/(*Q*_C_ + *Q*_PG_). Several datasets, however, only distinguish total grassland (PG + TG) from non-grass cropland (C − TG); in these cases we later estimated permanent grassland rates from total grassland rates (see details below).
- Gap-filling. Several data sources only report rates, not areas or quantities. In such cases, when the land category was known, we used the previously collected area data as necessary to calculate quantities, e.g., *Q*_C_ = *R*_C_*A*_C_. Furthermore, when rates and areas were reported for fertilized grassland (e.g., *R*_*PGf*_ and *A*_*PGf*_), we calculated the average rate on grassland using grassland areas from FAOSTAT/Eurostat (e.g., *R*_PG_ = *R*_*PGf*_*A*_*PGf*_/*A*_PG_).

#### Estimating the share of synthetic N fertilizer applied to cropland

In principle, the share of synthetic N applied to cropland can be calculated as *Q*_C_/*Q*_tot_ using a *Q*_C_ value from one of the above data sources and *Q*_tot_ from the IFA/FAOSTAT databases. Alternatively, if only *Q*_PG_ is known, the quantity to cropland can be estimated as *Q*_C_ = *Q*_tot_ − *Q*_PG_. However, this method is not ideal since it introduces noise and possibly bias stemming from the different methods used to estimate total fertilizer consumption and land category fertilizer use. We therefore designed the following method to estimate the share to permanent grassland, which uses the total IFA/FAOSTAT fertilizer quantities only as a last resort.

As a primary option, we used the following estimate. Assuming that all synthetic fertilizer is used on agricultural land (see Section “Synthetic N fertilizer consumption” above), the share applied to cropland can be expressed as

2 $$\frac{{Q}_{{\rm{C}}}}{{Q}_{{\rm{C}}}+{Q}_{{\rm{PG}}}}=\frac{{R}_{{\rm{C}}}{A}_{{\rm{C}}}}{{R}_{{\rm{C}}}{A}_{{\rm{C}}}+{R}_{{\rm{PG}}}{A}_{{\rm{PG}}}}={\left(1+\frac{{R}_{{\rm{PG}}}}{{R}_{{\rm{C}}}}\frac{{A}_{{\rm{PG}}}}{{A}_{{\rm{C}}}}\right)}^{-1}.$$

Note that this expression does not depend on the total fertilizer consumption (e.g., from IFA/FAOSTAT) but only on the area ratio *A*_PG_/*A*_C_ and the rate ratio *R*_PG_/*R*_C_. Whenever possible, we used this expression with area rate data from our own study and rate ratios calculated without mixing data from different publications. In the very few cases where different publications produced different rate ratio estimates for the same country/year, we used the average of the estimates.

A variant of this primary option was used in the cases where rates are available only for total grassland (PG + TG) and non-grass cropland (C − TG). To estimate the rate ratio *R*_PG_/*R*_C_ in these cases, additional data or assumptions are necessary. In some cases, we simply assumed that the average rate is equal on permanent and temporary grassland, but in other cases there was clear evidence of higher rates on temporary grassland. If the fertilizer rate on temporary grassland is *k* times the rate on permanent grassland, *R*_TG_ = *kR*_PG_, then

3 $${R}_{{\rm{PG}}+{\rm{TG}}}={R}_{{\rm{PG}}}\frac{{A}_{{\rm{PG}}}+k{A}_{{\rm{TG}}}}{{A}_{{\rm{PG}}+{\rm{TG}}}},$$

and similarly,

4 $${R}_{{\rm{C}}}=\frac{{R}_{{\rm{C}}-{\rm{TG}}}{A}_{{\rm{C}}-{\rm{TG}}}+k{R}_{{\rm{PG}}}{A}_{{\rm{TG}}}}{{A}_{{\rm{C}}}}.$$

It follows from Eqs. (3) and (4), using the shorthand *m* = *A*_PG+TG_/(*A*_PG_ + *kA*_TG_) and noting that *R*_PG_ = *mR*_PG+TG_, that

5 $$\frac{{R}_{{\rm{PG}}}}{{R}_{{\rm{C}}}}=\frac{m{R}_{{\rm{PG}}+{\rm{TG}}}{A}_{{\rm{C}}}}{{R}_{{\rm{C}}-{\rm{TG}}}{A}_{{\rm{C}}-{\rm{TG}}}+km{R}_{{\rm{PG}}+{\rm{TG}}}{A}_{{\rm{TG}}}}={\left(\frac{{R}_{{\rm{C}}-{\rm{TG}}}}{m{R}_{{\rm{PG}}+{\rm{TG}}}}\frac{{A}_{{\rm{C}}-{\rm{TG}}}}{{A}_{{\rm{C}}}}+k\frac{{A}_{{\rm{TG}}}}{{A}_{{\rm{C}}}}\right)}^{-1}.$$

Note that this expression, like Eq. (2), does not depend on the total quantity *Q*_tot_ but only on various rate ratios and area ratios.

As a secondary and last option, if only one of the rates was known, we instead estimated the rate ratio using the IFA/FAOSTAT total fertilizer quantity *Q*_tot_. For example,

$$\frac{{R}_{{\rm{PG}}}}{{R}_{{\rm{C}}}}=\frac{{R}_{{\rm{PG}}}{A}_{{\rm{C}}}}{{Q}_{{\rm{C}}}}=\frac{{R}_{{\rm{PG}}}({A}_{{\rm{tot}}}-{A}_{{\rm{PG}}})}{{Q}_{{\rm{tot}}}-{R}_{{\rm{PG}}}{A}_{{\rm{PG}}}},$$

where *A*_tot_ is the total agricultural land area. More generally, the rate ratio ${R}_{x}/{R}_{\widetilde{x}}$ between a land category *x* and its complement $\widetilde{x}$ (i.e., the remainder of the agricultural land) can be written as

6 $$\frac{{R}_{x}}{{R}_{\widetilde{x}}}=\frac{{R}_{x}({A}_{{\rm{tot}}}-{A}_{x})}{{Q}_{{\rm{tot}}}-{R}_{x}{A}_{x}}.$$

We used this equation in several cases (details below) but only when the rate ratio could not be calculated directly as the ratio of two rates.

As a final step, for years without data, we gap-filled the estimated rate ratios *R*_PG_/*R*_C_ using linear interpolation between data points and constant extrapolation before and after the first and last data point. We then estimated the share applied on cropland using Eq. (2) with area data (*A*_C_ and *A*_PG_) from FAOSTAT/Eurostat. We finally calculated the fertilizer quantities to cropland and permanent grassland using these shares so that the quantities agree with total fertilizer consumption statistics but not necessarily with the collected data on crop-specific rates and quantities. The resulting estimates of synthetic N application on cropland and permanent grassland are provided in the data record^12^.

#### Countries not using synthetic N fertilizer on permanent grassland

Based on the FAO/EFMA data and other publications we concluded that the following countries have zero or negligible fertilizer rates to permanent grassland: Bulgaria, Croatia, Estonia, Finland, Greece, Hungary, Latvia, Lithuania, Portugal, Romania, Spain, and Sweden. These countries can conceptually be divided into two groups. One group is the countries in north-east Europe (Sweden, Finland, Estonia, Latvia, Lithuania) which all have substantial areas of temporary grassland. In Sweden and Finland, intensive grassland cultivation is a central part of agriculture but occurs almost exclusively on arable land^92,93^. In Estonia, Latvia, and Lithuania, the grassland cultivation practices have varied substantially through the decades; since 1992 much grassland has been abandoned or very extensively managed^57,77,94,95^, and the FAO/EFMA datasets^87,90^ suggest that if any synthetic N inputs have been applied to grassland, it has been to temporary grassland. The other group (Bulgaria, Croatia, Greece, Hungary, Portugal, Romania, and Spain) has small or negligible areas of temporary grassland but considerable areas of permanent grassland, which however are mostly extensively managed due to a combination of economic and climatic conditions^17,77,96–103^. For both groups, the quantitative data^87–90^ suggests that synthetic N inputs to permanent grassland was negligible at least during the 1990s and 2000s, and since no data could be found before the 1990s we have set it to zero for the whole period 1961–2019.

A caveat to this assumption applies especially to the former communist states, which before the 1990s typically had much higher fertilizer inputs and different patterns of agricultural land use. In this study this is not an issue for Croatia, Estonia, Latvia, and Lithuania, which are included only from 1992, but for the former socialist republics of Bulgaria, Hungary, and Romania, which are included from 1961, we emphasize that data are very scarce.

#### Countries using synthetic N fertilizer on permanent grassland

**Austria**. Most of Austria’s permanent grasslands are unfertilized^104^. Available quantitative data^87–90,105^ show that the rate ratio *R*_PG_/*R*_C_ has been fairly constant in the period 1993–2006. Intensification of grassland fertilization is reported to have occured in the 1970s–1980s^104^ which roughly coincides with the steepest increase in total synthetic fertilizer use in Austria. We therefore extrapolated the average of the available rate ratio (*R*_PG_/*R*_C_ ≈ 0.11) to the whole period 1961–2019.

**Belgium and Luxembourg**. Belgium has a long history of heavy grassland fertilization^106^ but quantitative data are surprisingly scarce. The FAO/EFMA data shows that the average synthetic N rate on grassland (PG + TG) was on average 20% higher than on non-grass (C − TG) in the 1990s and 2000s. Since the fertilizer rates in Belgium-Luxembourg^88^ are practically indistinguishable from those referring to Belgium alone^87,89,90^, we pooled all the data and assumed the same rate ratio *R*_PG_/*R*_C_ for Luxembourg (<10% of the combined agricultural area). Before the 1990s we have only found few rough estimates of average grassland fertilization during the 1970s^91,107^ and these suggest average grassland fertilization at or above the level of cropland fertilization. One publication from 1955 calls grass Belgium’s “main crop” and discusses annual synthetic N application rates in the range 0–160 kg N ha^−1 ^^108^, which suggests that grasslands were fertilized with similar rates as cropland already in the 1950s. We extrapolated the 1990s–2000s average rate ratio (*R*_PG+TG_/*R*_C−TG_ ≈ 1.2) to the whole period 1961–2019 before estimating *R*_PG_/*R*_C_ using Eq. (4) assuming *R*_PG_ = *R*_TG_ (i.e., *k* = 1).

**Czechia, Slovakia, and former Czechoslovakia**. Grasslands in Czechia and Slovakia are almost exclusively permanent and much of them situated on poorer soils in upland and mountain regions^109–111^. Between year 2000 and 2006 the rate ratio *R*_PG_/*R*_C_ fell from about 0.12 in Czechia and 0.09 in Slovakia to almost zero in both countries^89,90^. Lacking data on the situation in former Czechoslovakia, we calculated the combined rate ratio *R*_PG_/*R*_C_ ≈ 0.10 for Czechia and Slovakia in 1993 and extrapolated it to Czechoslovakia 1961–1992.

**Denmark**. Denmark’s grasslands are about equal parts temporary and permanent and both categories have been fertilized to varying degrees. Interpretation of the few available data is somewhat involved since some data refer to permanent grassland and others to total grassland. Specifically, three of the FAO/EFMA datasets^88–90^ show that the rate ratio *R*_PG_/*R*_C_ fell from about 0.70 to 0.17 between 1996 and 2006. An expert estimate from 1980^91^ is that permanent grasslands were then fertilized with 150 kg N ha^−1^y^−1^ which translates using Eq. (5) to *R*_PG_/*R*_C_ ≈ 1.15. The remaining two data points, one for 1966^112^ and one for 1993^87^, concern total grassland. To estimate the rate to permanent grassland in these cases we used Eq. (4) with *k* = 1.5 since several data sources^88–90,112^ suggest that temporary grasslands have on average received perhaps 50% higher synthetic N rates than permanent.

**France**. France has large areas of both permanent and temporary grasslands which both have received synthetic fertilizer to varying degrees since the 1950s. This development has been quantified in several national surveys. We used rate ratios *R*_PG_/*R*_C_ based on these national surveys as compiled by Le Noë *et al*.^5^.

**Germany**. According to the FAO/EFMA data for Germany^87–90^, the rate ratio *R*_PG_/*R*_C_ decreased from about 0.8 in 1993 to 0.6 in 2006. The two earliest FAO/EFMA datasets^87,88^ seemingly refer to total grassland and we therefore estimated *R*_PG_/*R*_C_ using Eq. (4) with *k* = 1; the estimation is insensitive to the exact value of *k* since only about 4% of the grassland was temporary at the time.

Before the 1990s only a handful of estimates are available for West and East Germany. In West Germany, synthetic N fertilization in 1965 was reported at 17 kg N ha^−1^y^−1 ^^113^. In 1971 and 1979, average rates in West Germany of 79 and 88 kg N ha^−1^y^−1^ can be calculated from rate estimates^91^ for hay meadows (3/5 of West German grassland^114^) and pastures (2/5 of the grassland). In East Germany, anecdotal evidence from 1977^115^ is that around 100 kg N ha^−1^y^−1^ was used on grassland. However, since West Germany accounted for around 80% of the total grassland area we estimated the rate ratio *R*_PG_/*R*_C_ in 1965, 1971, and 1979 using Eq. (5) with the above-mentioned West German rate estimates applied to the combined permanent grassland area of West and East Germany.

**Ireland**. Permanent and temporary grassland covers about 85% of Ireland’s agricultural area and receives a similar share of the synthetic N inputs. We used data from national fertilizer surveys in 1973, 1985, 1995, and 1999–2015^116–121^. These surveys report the average rates applied on most arable crops and on grassland and we used these to calculate area-weighted rates on grassland and non-grass cropland. Some crop areas missing from the surveys were filled using data from Eurostat^28^ and the Central Statistics Office of Ireland^122–125^. Since these surveys refer to grassland excluding rough grazing, we recalculated the fertilizer quantities to average rates on the FAOSTAT/Eurostat total grassland areas used in this study. For the years 2005–2008 where two surveys^120,121^ slightly disagree on fertilizer quantities and areas, we used the average of the resulting rate ratios *R*_PG+TG_/*R*_C−TG_. For the 1973 data^116^ we could not find the grassland areas and instead estimated the grass/non-grass application rate ratio from the non-grass rate *R*_C−TG_ using Eq. (5). All the data concern grass vs. non-grass and we estimated the rate ratio *R*_PG_/*R*_C_ using Eq. (4) assuming *k* = 2 to reflect the higher average production intensity on temporary grassland^116,118,126^.

**Italy**. In Italy, the FAO/EFMA datasets^87–90^ show that the rate ratio *R*_PG_/*R*_C_ has been fairly stable in the period 1993–2006. We extrapolated the average rate ratio *R*_PG_/*R*_C_ ≈ 0.13 to the whole period 1961–2019.

**Netherlands**. In the Netherlands, synthetic N inputs to grassland increased from some 20 kg N ha^−1^y^−1^ in the 1940s to a peak above 250 kg N ha^−1^y^−1^ in the 1980s before decreasing to about 150 kg N ha^−1^y^−1^ during the 2000s. This development can be fairly well quantified by combining several data sources. For the period 1980–2008, we used data compiled for the Dutch National Emission Model for Agriculture^127^ to calculate the rate ratio *R*_PG+TG_/*R*_C−TG_. In 1970, a combination of expert estimates^128^ and a national survey on grassland fertilization^129^ show that *R*_PG+TG_/*R*_C−TG_ ≈ 1.5. Before 1970, based on grassland fertilizer rates *R*_PG+TG_ from several sources^129–134^ and using Eq. (5) we estimated a linear increase from *R*_PG+TG_/*R*_C−TG_ = 1 in 1961. We then estimated *R*_PG_/*R*_C_ using Eq. (4) with *k* = 1 since the data do not show any clear differences in fertilizer rates between temporary and permanent grassland^87–89,127^.

**Poland**. In the period 1993–2006, the FAO/EFMA datasets^87,89,90^ show a roughly constant rate ratio *R*_PG_/*R*_C_ ≈ 0.6. Before the 1990s we have not found quantitative data, although an increasing trend of synthetic N inputs on permanent grassland was noted already in 1965^62^ and permanent grassland productivity then increased along with overall synthetic N inputs into the 1970s and 1980s^135^. We therefore extrapolated the average 1993–2006 rate ratio to the whole period 1961–2019.

**Slovenia**. Slovenian grasslands are predominantly permanent. In the period 2006–2012, national statistics^136^ show a stable rate ratio *R*_PG_/*R*_C_ ≈ 0.37 which we extrapolated to the whole period 1992–2019.

**United Kingdom**. The United Kingdom has Europe’s longest and most complete dataset of synthetic fertilizer inputs to grassland and crops. Partial surveys of England and Wales started in the early 1940s^137^ and have continued in various forms ever since^138–141^. Annual time series of fertilizer use on permanent and temporary grassland as well as other crops are available for England and Wales since 1969 and for Great Britain since 1982. Northern Ireland is not included in the annual surveys.

Two main complications arise in the estimation of the United Kingdom’s rate ratio *R*_PG_/*R*_C_ from these data. The first is that Scotland (about 12–15% of the synthetic N use^141^) is not included until 1982 and Northern Ireland (4–7% of total synthetic N use) is not included at all. The second is that the surveys refer to permanent grassland excluding rough grazing, the area of which is not always given in the data, and therefore we were unable to calculate the total quantity or average rate applied on the total permanent grassland area. We addressed these issues as follows. First, since the trends in fertilizer rates have been very similar between Great Britain and England and Wales, we used the historical rates *R*_C−TG_ and *R*_TG_ from England and Wales, adjusted down for the somewhat lower average application rates in Great Britain (*R*_C−TG_ about 2% lower and *R*_TG_ about 2.5% lower). We then calculated area-weighted average rates *R*_C_ using FAOSTAT/Eurostat areas *A*_C−TG_ and *A*_TG_ for the whole United Kingdom. Finally, we used these *R*_C_ values and the United Kingdom’s total fertilizer quantities to estimate the rate ratio *R*_PG_/*R*_C_ using Eq. (5).

Most of the needed data 1970–2017 are available in a spreadsheet compilation from DEFRA^141^. Data for 2018–2019 as well as temporary grassland rates 1992–2019 are found in the annual survey reports^142–169^. Data from 1962, 1966, 1966, and 1969, as well as temporary grassland rates from the 1970s have been published elsewhere^138,139^. We filled a data gap in the temporary grassland rates *R*_TG_ in England and Wales 1977–1991 by multiplying those years’ rates to permanent grassland (excluding rough grazing) by the 1970–76 and 1992–2000 average ratio between these two rates. This rate ratio was 1.85 in that period and has been fluctuating around 1.9 during the whole period 1957–2019.

### Manure input to cropland

Manure N quantities excreted by livestock were estimated by Lassaletta *et al*.^17^. These estimates account for the changes in livestock productivity over time, which is a crucial consideration since the excretion per head of livestock has changed very substantially in some categories since 1961. We estimated manure excretion using the same method but using the latest FAOSTAT livestock data.

This section describes a new estimate of the manure N flows after excretion aiming primarily to estimate the share of the excreted N applied to cropland.

We considered two pathways for manure N input to cropland: (1) excretion in animal houses followed by storage and field application, and (2) excretion of grazing animals on cropland (temporary grassland and aftermath/stubble grazing). The main steps of the calculation, described in further detail below, are:

1. Allocation of excreted N to different manure management systems, including excretion on pasture.
2. Estimation of N losses in houses and storage.
3. Allocation of managed manure N and grazing N excretion to cropland:
  - The share of managed manure N applied to cropland (including temporary grassland).
  - The share of grazing N excreted on cropland.

The resulting manure N flows are available in the data record^12^.

#### Allocation of excreted N to manure management systems

National time series of N excretion in different manure management systems (including N excretion on pasture) are reported annually as part of the national greenhouse gas inventories of Annex I parties to the United Nations Framework Convention on Climate Change (UNFCCC)^170^. We used these data, with minor adjustments as explained below, to allocate the excreted N to manure management systems. We compiled manure management data from Table 3.B(b) of the 2020 submissions in the Common Reporting Format (each year’s submission reports a time series starting in the late 1980s or 1990) for the 26 present-day countries^12^. The data start in 1990 for most countries, and up to a few years earlier in the following countries: Hungary (1985), Slovenia (1986), Bulgaria (1988), Poland (1988), and Romania (1989). We calculated excretion data for Czechoslovakia (1990–1992) and Belgium-Luxembourg (1990–1999). As the 2020 submissions only cover the period up to 2018, we extrapolated 2018 results to 2019. We also aggregated the UNFCCC manure management systems and livestock classes to a simplified nomenclature as shown in Table 6 and Table 7.

**Table 6:** Aggregation of UNFCCC manure management systems.

Simplified classes	UNFCCC classes
Grazing	“Pasture range and paddock”
Solid	“Composting”, “Daily spread”, “Solid storage and dry lot”
Liquid	“Anaerobic lagoon”, “Digesters”, “Liquid system”
Other	“Other”

Also available in machine-readable form in the data record^12^.

**Table 7:** Aggregation of UNFCCC livestock classes.

Simplified classes	UNFCCC classes
Ruminants and equines	“Cattle”, “Sheep”, “Buffalo”, “Goats”, “Horses”, “Mules and Asses”
Pigs	“Swine”
Poultry and rabbits	“Poultry”, “Rabbit”

Also available in machine-readable form in the data record^12^.

The resulting quantities were converted into shares excreted in the different manure management systems for each of the livestock classes. We extrapolated these shares as necessary back in time to 1961. The N quantities excreted in houses and on pasture were then calculated by multiplying the appropriate shares by total N excretion, aggregated to the same livestock classes.

#### Estimation of N losses in houses and storage

The share of excreted N lost to the environment from livestock houses and manure storage is governed by a long list of different factors, including house design and cleaning systems, storage system and type of cover, the length of the storage period, the climate, and the composition of the manure^171^. Modeling efforts have now advanced so that several of these factors can be accounted for in estimates of N losses from manure management systems. However, for the historical perspective taken here, data are lacking for all but the most basic of the determining parameters. Therefore, in the global study by Lassaletta *et al*.^17^, a generic 30% loss rate was assumed between excretion and field application. In this European study, we instead use constant country-specific estimates of N loss shares in housing and storage in year 2000 established by Oenema *et al*.^172^. This dataset covers the former EU27 countries, i.e., all the countries except Croatia, for which we assumed the same loss rate as for Slovenia. For Czechoslovakia and Belgium-Luxembourg, we calculated excretion-weighted averages from their present-day constituents (back-calculated from the per-hectare rates of the paper using the Eurostat Utilised Agricultural Area). The N loss shares estimated for year 2000 by Oenema *et al*.^172^ are quantitatively in line with a more recent systematic review by Pardo *et al*.^173^.

#### Allocation of stored and grazing manure between cropland and permanent grassland

To estimate application of stored manure to cropland and permanent grassland, we used country-specific expert estimates of the shares of stored manure applied to grass and non-grass. These data were collected through questionnaires in 1997/1998 and in the early 2000s^174,175^. For pigs and cattle, separate estimates are given for liquid and solid manure, while for poultry the estimates are totals across manure management systems. Separate estimates for pigs and cattle are given for the Netherlands, Slovakia, Sweden, and the UK; for the remaining countries the land allocations of liquid and solid manure concern the sum of pig and cattle manure. Since the land allocation data do not cover all livestock types separately, we used the cattle data for all ruminants and equines, pig data for pigs, and poultry data for poultry and rabbits.

For poultry and rabbits, we calculated the manure N applied to grassland and non-grass simply by multiplying the post-storage manure N quantities by the corresponding land allocation shares. However, for pigs and ruminants and equines, with different land allocations for liquid and solid manure, the manure management system shares must be accounted for. For this, we used average shares during 1997–2001 to match the time frame of the land allocation data.

The binary division between liquid and solid manure management assumed in the land allocation data causes a mismatch with the UNFCCC manure management data, where countries sometimes use the manure management category “Other” (see Table 6). However, in all but three countries, the “Other” category has a minor share and we ignored the mismatch, calculating a partition between liquid and solid in proportion to their reported shares (i.e, so that they add up to 100%). In three countries, the “Other” category accounted for more than 20% of in-house N excretion: Hungary (23%), Ireland (99%), and Spain (33%). For these countries we instead compiled data on liquid and solid shares from official country reports to the Convention on Long-Range Transboundary Air Pollution (CLRTAP)^176–178^. Having established the liquid/solid division for cattle, we calculated the average 1997–2001 shares of stored manure applied on grassland and non-grassland.

For the countries not included in the land allocation dataset, we extrapolated grass/non-grass shares from other countries: from Austria to Slovenia; from Lithuania to Estonia and Latvia; and from Slovakia to Bulgaria, Croatia, and Romania. For poultry and rabbits only, results were extrapolated from Belgium to the Netherlands and Luxembourg, and from Finland to Sweden. For Belgium-Luxembourg and former Czechoslovakia, we calculated excretion-weighted averages from their present-day constituents.

For Portugal, we disregarded the reported allocation of pig and cattle manure^175^ which suggests that 80% is discharged outside agriculture. This value appears unlikely at first sight and moreover directly contradicts a 2009 survey from Statistics Portugal^179^ according to which the overwhelming majority of stored manure is used in agriculture. We instead extrapolated allocation shares from Spain to Portugal.

We then extrapolated the resulting shares of stored manure applied to grassland and non-grassland to the whole time period, based on the assumption that the manure N allocation between grass and non-grass crops has remained constant although manure management systems have varied in the past. The resulting allocation of stored manure is available in the data record^12^.

As a final step, following Le Noë *et al*.^5^ we divided manure N inputs to grassland (i.e., all the grazing excretion and the share of manure applied to grassland) between permanent and temporary grassland in proportion to their areas. The input to cropland is the sum of input to temporary grassland and non-grassland crops.

### Final compilation of data collections and results

The collected literature data, inspected figures, and final results were finally compiled into the data records listed below.

## Data Records

All the input data and final results, as well as the source code and numerous intermediate results and figures, have been publicly archived in a data record^12^ in the research data repository Figshare (https://figshare.com). The data record contains the following:

### Main results

#### Data tables (csv files, accompanied by text files with metadata)

- Cropland N budget terms:

–Cropland N harvest
–Symbiotic N fixation
–Synthetic N to cropland
–Manure N to cropland
–Atmospheric N deposition to cropland

- Land areas:

–Cropland (from FAOSTAT)
–Permanent grassland (from FAOSTAT)
–Crop area sum (sum of our crop categories)
–Cropland in use (minimum of cropland and crop area sum)

- Crop production by crop category:

–Area
–N harvest
–N yield

- Crop production by crop, fodder crops only:

–Area
–N harvest
–N yield

- Symbiotic N fixation by crop category
- Synthetic N fertilizers:

–Applied quantities (total, cropland, permanent grassland)
–Average application rates (cropland, permanent grassland)

- Manure intermediate results:

–Excretion by livestock class
–Destination shares for stored manure (cropland/grassland)
–Shares of in-house excretion lost from houses and during storage

- Manure N flows (country totals):

–excreted total
–excreted in house
–excreted grazing
–excreted grazing on cropland
–excreted grazing on permanent grassland
–lost from houses and storage
–applied to cropland
–applied to permanent grassland

#### Figures (png files)

- Crop categories time series per country: N harvests and areas
- Cropland N budget time series per country: quantities, average rates kg N ha^−1^y^−1^ cropland in use

### Input data, source code, and output data

In addition to the main results mentioned above, the data record contains

- The input data, including the literature data compilations mentioned in this paper.
- Python source code to reproduce the results from input data.
- Output data, in addition to the main results, for example many of the intermediate results and hundreds of figures used to inspect and validate the data processing steps.

## Technical Validation

The dataset presented in this paper builds on careful inspection and cross-comparison of multiple data sources. To the extent possible, all calculation steps have been automated using Python scripts, but the basis of the work has consistently been visual inspection and selection of the most appropriate data from available alternatives. The method section above describes in detail the steps we have taken to guarantee the consistency, completeness, and accuracy of the results.

On a more general level, the technical validation in this study can be described as standing on three pillars:

1. Internal consistency has been a guiding principle throughout the process. We have carefully checked or defined variables to achieve internal consistency, e.g., agreement of total fertilizer consumption and application to cropland and permanent grassland; agreement of crop category harvest = area × yield; etc.
2. Cross-checking of data against multiple independent sources where possible, e.g., only using crop-specific fertilizer application data if totals agree with national totals; cross-checking cropland and grassland areas between FAOSTAT and Eurostat databases; comparing and hand-selecting fertilizer quantities and crop area data from multiple sources, etc.
3. Detailed scrutiny of visualizations of all included data and of intermediate and final results of calculations. This approach has been invaluable to identify and address data gaps and errors. The text of the method section as well as the figures in the data record^12^ give concrete details on key aspects of this work.

## Usage Notes

All the results tables in the data record^12^ are provided as csv files which can be read using many spreadsheet applications and data processing languages. The tables are provided in so-called “tidy format”^180^ which facilitates machine reading and further processing.

The files with literature data compilations and intermediate results files are provided in csv and/or Excel (xlsx) format. Most of them conform to tidy data principles, although some conform to the format encountered in the source publication. The compilations of literature data on fodder crop areas and crop-specific N inputs are provided in Excel format which users may find convenient for inspection and editing. In addition, these literature data compilations are also provided as csv files with identical contents, for maximum compatibility and to facilitate machine reading.

## Data Availability

Source code in Python for the data processing is available in the data record^12^.
